# Yogan Pillay: integrating tuberculosis and HIV care in South Africa

**DOI:** 10.2471/BLT.18.030518

**Published:** 2018-05-01

## Abstract

South Africa has the highest burden of HIV-associated tuberculosis. Yogan Pillay tells Fiona Fleck how the health ministry has brought the two programmes and services together.

Q: How did you become interested in public health?

A: I’ve been politically active since I was at university in the 1980s. When I started working as a clinical psychologist in a hospital, the first thing that struck me was the inequities. That was during the apartheid era when there were serious inequities between white and black – some of which continue to this day – but back then they were institutionalized. You saw the inequities in many ways. I was paid less than the other students in my master’s class, who were white. In the wards, I saw how more resources were expended on white psychiatric patients, compared to black ones. I was highly politicised and spent my weekends working as a paralegal with the unions supporting workers and teaching them English as a second language, so I have a long history of engagement in the anti-apartheid movement.

Q: What were your early experiences in public health?

A: As a clinical psychologist I knew that seeing individual patients was important, but I had a strong desire to do something to improve health on a population level. I won a scholarship to do a PhD in public health at Johns Hopkins, where I wanted to work with Vincente Navarro, who had fled Spain during the Franco era and who was the editor-in-chief of the *International Journal of Health Services*. When I returned to South Africa in 1995, a year after the transition to democracy, the new department of health was being established and I was asked to apply for a job there.

Q: In 2003, the South African government started to provide antiretrovirals (ARV) for people with human immunodeficiency virus (HIV) infection. How has the epidemic developed since then?

A: We estimate that 7.1 million people (12.6%) are infected with HIV out of a population of 56 million (2016 figures). Every year, we have an estimated 260 000 new infections. Adolescent girls and young women aged between 15 and 20 years are worst affected: every week there are nearly 2000 new infections in this age group. The vectors in this sexually transmitted generalized epidemic are men. That’s HIV in South Africa in a nutshell. By 2009, we managed to initiate about 400 000 patients on ARVs. Today it is 4.2 million and our target is 6.1 million on ARVs by December 2020. In 2004, 30% of pregnant mothers with HIV infection passed on the infection to their infants, today most of these mothers are on ARVs and only 1.1% of their babies are infected at 10 weeks postpartum. So we have made some progress.

Q: In 2004, South Africa declared tuberculosis a national emergency. How does the epidemic look today?

A: According to the World Health Organization (WHO) estimate, we should be finding around 430 000 people with tuberculosis every year. But we record about 260 000 notifications per year – which is down from over 400 000 in 2009. We think that the tuberculosis numbers have been coming down because we are able to prevent and treat tuberculosis better. That is, we are putting large numbers of people with HIV on isoniazid to prevent them from contracting tuberculosis. Two, people with HIV are also less likely to contract tuberculosis because they are receiving ARVs earlier in the course of their disease. Three, our tuberculosis cure rates have improved. However, 60% of new cases of tuberculosis are in people living with HIV infection. We have managed to get this down from 80% a few years ago, but the high co-infection rate is still very worrying.

Q: Why? 

A: The high co-infection levels are worrying because tuberculosis is the cause of death for most people who die due to complications of HIV infection. This means that to reduce deaths in people with HIV, we must prevent them from contracting tuberculosis. This will also help us decrease mortality from tuberculosis in general.

Q: How have you been addressing the burden of HIV-associated tuberculosis?

A: We have been treating tuberculosis and HIV infection as two sides of the same coin. In 2009, we started to decentralize HIV treatment from hospitals to primary health care. At that time, we were already treating tuberculosis at the primary health care level and so we started to treat HIV at that level as well. In 2014, WHO, the United Nations Children’s Fund and other partners did an external review of our HIV–tuberculosis programme that showed that while integration of these services had taken place in general, there were still gaps in the way integration was happening and some patients were falling between the cracks.

Q: Few countries with a high burden of this co-infection have integrated HIV and tuberculosis services. Is it particularly challenging? How does such integration of HIV and tuberculosis services look in reality?

A: Integration means several things. Either HIV and tuberculosis services are provided by the same nurse. Or, they are provided in different consulting rooms in the same health centre by different providers – we call this the supermarket approach. In addition, at the district level, we have HIV, sexually transmitted infections and tuberculosis managers who support and reinforce the integration of these three sets of services. Having a single supervisor or mentor at district level helps to facilitate integration at the health facility level.

“Treatment is free at the point of care, but there are still many barriers to ensuring that patients get the treatment they need.”

Q: Does integration for HIV and tuberculosis services happen in all district health facilities?

A: It is rare for integration not to happen, but it may occur when the nurse trained to initiate people on ARV is not confident, doesn’t have the support to see patients with tuberculosis or has not been trained yet. We have a lot of staff rotation, so some health facilities may lose the nurses who attend to patients with both infections. At the health ministry, we closely monitor efforts to bring these programmes together at district level. When our ministry reviews the tuberculosis and HIV programmes, we do this jointly. When the two teams from the national department monitor and support efforts on the district level, they visit the districts together. 

Q: Are you satisfied with the progress you are making in bringing HIV and tuberculosis programmes and services together? 

A : It is far from perfect because there are still patients with tuberculosis who are not being tested and treated for HIV, or HIV patients who are not being screened for tuberculosis at some of our facilities. However, it has also been a very positive experience. It means that our district health services no longer focus on one or two symptoms or on individual cases of disease, but on the whole person and that person’s overall wellbeing, so it’s a person-centred approach. Treatment is free at the point of care, but there are still many barriers to ensuring that patients get the treatment they need, including low levels of literacy and numeracy. This relates to the poor education black people received during apartheid and still receive in township schools. This affects patients as well as health workers. 

Q: How are you addressing these barriers to deliver services?

A: We are trying to help patients as much as possible, using digital communications, as more than 90% of our population has access to a mobile phone. We have about two million pregnant women who receive text messages on what to expect at every stage of their pregnancy and during the baby’s first 12 months of life. Messages on HIV and tuberculosis are also included in the weekly messages sent to pregnant women who register on Momconnect. Patients can also use this system to report problems and complaints about the services they are receiving. We now do something similar for nurses, who sometimes feel unsupported by their management. We have about 22 000 nurses signed up. For health workers, there are two types of message: technical (on how to improve skills) and motivational (as they are working under huge pressure). We also do patient satisfaction surveys and we have several very energetic nongovernmental organizations. This includes the Treatment Action Campaign – with their own people on the ground, who report challenges such as treatment that is not respectful towards patients, drug stockouts and long waiting times.

Q: Are there other models of service delivery in other countries that you are looking at?

A: Yes, we have been looking at Brazil’s primary health care approach, Ethiopia’s community-based health extension programme and Zimbabwe’s prevention of HIV infection initiatives. We are moving towards universal health coverage, called National Health Insurance, and my other job is to finalize the service packages for this. Here, we are learning from Thailand and its universal health coverage system, as well as from the National Institute for Clinical Excellence in the United Kingdom of Great Britain and Northern Ireland about health technology assessment and how to do cost-effectiveness studies to decide how to get the best value for our money. Other countries are learning from us too. For example, we recently had a large group from Thailand visiting us to learn about our HIV response and our laboratory system.

“As a consequence of putting so many people on ARVs and treatment for tuberculosis, they are living longer and ageing. So we are addressing a growing burden of noncommunicable diseases.”

Q: How are you applying what you have learned from integrating HIV and tuberculosis services to other priorities such as noncommunicable diseases?

A: We are finding that as a consequence of putting so many people on ARVs and treatment for tuberculosis, they are living longer and ageing. So we are addressing a growing burden of noncommunicable diseases, including hypertension, diabetes and obesity. We have found that it helps to offer a comprehensive package of interventions, and this is something we are starting to do as we move towards universal coverage of health services. That way, we can take more of a health systems approach, rather than thinking in silos, and respond better to patients’ needs across the life cycle, including health promotion, prevention, treatment and palliation. We are only just beginning. Our training of health-care workers has to be more patient focused and more primary care focused. These are the kinds of things that the whole world is struggling with, so we know we are not alone.

